# Histological Evaluation for Collagen Expression Prior to LVAD Implantation Is Useful to Estimate Weaning Success

**DOI:** 10.3390/biomedicines13071515

**Published:** 2025-06-20

**Authors:** Maja-Theresa Dieterlen, Lea Schreiber, Kristin Klaeske, Joanna Jozwiak-Nozdrzykowska, Michael A. Borger, Alexey Dashkevich, Sandra Eifert, Michal Nozdrzykowski

**Affiliations:** 1 Department of Cardiac Surgery, HELIOS Clinic, Heart Center Leipzig, University Hospital Leipzig, 04289 Leipzig, Germanymichael.borger@helios-gesundheit.de (M.A.B.); alexey.dashkevich@helios-gesundheit.de (A.D.); michael.nozdrzykowski@helios-gesundheit.de (M.N.); 2 Department of Cardiology, Heart Center Leipzig, Leipzig University, 04289 Leipzig, Germany

**Keywords:** collagen, left ventricular assist device, weaning, recovery, histology

## Abstract

**Introduction:** The implantation of a left ventricular assist device (LVAD) is a life-saving therapeutic option for patients with advanced heart failure. The treatment goal has to be determined prior to LVAD implantation. However, prognostic evaluation for defining the treatment goal could be improved for a time- and cost-effective medical treatment. **Methods:** Our study comprised seven patients who were weaned from LVAD (recovery group) and a control group without weaning (non-recovery group; *n* = 7). Myocardial tissue was analysed for connective tissue content by Masson–Goldner trichrome staining and for collagen I and collagen III expression by quantitative real-time PCR and immunohistochemistry. **Results:** The histological evaluation revealed comparable values for the percentage of total connective tissue (non-recovery: 46.3% [95% CI: 15.9–76.7], recovery: 43.4% [95% CI: 13.7–73.2], *p* = 0.43). mRNA expression analysis for collagen I and III expression could not detect a difference in collagen I (*p* = 0.16) and collagen III expression (*p* = 0.12) between the non-recovery and the recovery group. Immunohistochemical staining revealed that the percentages of collagen I (*p* = 0.05) and of collagen III (*p* = 0.01) were reduced in patients who do not recover compared to patients who recover under LVAD support. **Conclusions:** Our data indicate that histological evaluation for collagen expression prior to LVAD implantation could detect differences in the collagen content that could be helpful for estimating the weaning success.

## 1. Introduction

The implantation of a left ventricular assist device (LVAD) is a life-saving therapeutic option for patients with advanced heart failure. The treatment goal has to be determined prior to LVAD implantation and comprises the bridge-to-recovery, bridge-to-transplantation, bridge-to-candidacy and destination therapy. Nevertheless, the prognostic evaluation for determining the treatment goal could be enhanced to achieve a more time- and cost-effective medical treatment.

The prediction of the hemodynamic and clinical stability following potential LVAD withdrawal remains challenging, and consequently, the determination of treatment goals [1,2]. Patients diagnosed with non-ischemic cardiomyopathy, who are younger in age and have experienced a shorter duration of heart failure, have been observed to demonstrate a higher incidence of cardiac recovery [1]. A clinical assessment for successful recovery may include echocardiography on full, reduced or temporary withdrawal of LVAD support [2]. However, the reliability of this method as a predictor of weaning success remains to be ascertained. Consequently, the selection of additional parameters is imperative. As demonstrated by transcriptomic profile analysis, which revealed that myocardial recovery is associated with an increased expression of extracellular matrix (ECM) proteins, such as collagen I and III, in successfully weaned patients [3], a potential target for improved selection of the LVAD treatment goal is available.

We investigated in a small study cohort if the histological evaluation for collagen expression prior to LVAD implantation is useful for estimating weaning success.

## 2. Methods

### 2.1. Study Groups and Clinical Characteristics

The Ethics Committee of the Medical Faculty of the University of Leipzig, Germany, approved this study (ID: 240/16-ek, date: 17 October 2017), which was performed according to the guidelines of the Declaration of Helsinki (2013). Written informed consent was obtained from all patients.

The study comprised 14 patients with dilative cardiomyopathy who underwent an LVAD implantation between December 2016 and March 2021. Seven patients recovered within 17 months and were weaned from LVAD (=recovery group). We matched patients for a control group (=non-recovery group; *n* = 7) regarding sex, age at LVAD implantation and body mass index (BMI). Patients of the non-recovery group were not weaned from LVAD support. Patient’s characteristics such as gender, age and body mass index (BMI) as well as clinical data including left ventricular ejection fraction (LVEF), N-terminal pro b-type natriuretic peptide (NT-proBNP), NYHA class, comorbidities, kidney function parameters and nicotine abuse were recorded.

### 2.2. Cardiac Tissue Preparation

Myocardial tissue was obtained during the coring step for pump placement at the left ventricular apex during LVAD implantation. One part of the obtained myocardial tissue was fixed in 4% formaldehyde/phosphate-buffered saline (PBS; 137 mM NaCl, 2.7 mM KCl, 10 mM Na_2_HPO_4_, 1.8 mM KH_2_PO_4_, pH 7.4) for histological analysis. The second part of the myocardial tissue was dissected and snap-frozen in liquid nitrogen for polymerase chain reaction.

### 2.3. RNA Isolation and cDNA Synthesis

RNA isolation and cDNA synthesis of snap-frozen myocardial tissue for quantitative real-time polymerase chain reaction (qRT-PCR) were performed as described previously [4]. RNA was isolated using TRIzol reagent (Thermo Fisher Scientific Inc., Waltham, MA, USA) and chloroform. Following isolation, the complementary desoxyribonucleic acid (cDNA) was transcribed from RNA using the Applied Biosystems™ High Capacity cDNA Reverse Transcription Kit (Thermo Fisher Scientific Inc.) according to the manufacturer’s instructions. The purity and concentration of RNA and cDNA was determined using the Infinite 200 PRO microplate reader and the i-control™ 1.12 software (both Tecan Trading AG, Männedorf, Switzerland).

### 2.4. Quantitative Real-Time Polymerase Chain Reaction

qRT-PCR was performed as described previously [4]. For the quantification of mRNA expression of collagen I, collagen III and the housekeeping gene ribosomal protein L4 (RPL4), the QuantiNova SYBR^®^ Green PCR Kit (Qiagen, Hilden, Germany) was used. For each qRT-PCR reaction, 6.2 µL DEPC-treated water, 1.4 µL forward primer, 1.4 µL reverse primer, 10 µL SYBR Green PCR Master Mix and 1 µL cDNA were mixed. All reactions were performed in duplicate. The following primers were used: collagen I forward 5′-GCCATCAAAGTCTTCTGCAACA-3′, reverse 5′-CCGAACCAGACATGCCTCTT-3′; collagen III forward 5′-TGGGGTCAAATGAAGGTGAATTC-3′, reverse 5′-TGTTTTGCTCCATTCCCCAGT-3′; RPL4 forward 5′-CCAGGGTGCTTTTGGAAAC-3′, reverse 5′-AGATGGCGTATCGTTTTTGG-3′. Cycling was performed for 2 min at 95 °C, 10 s at 95 °C, 20 s at 60 °C for 45 cycles using the Light Cycler^®^ 480 II (Roche Molecular Systems Inc., Basel, Switzerland). The expression of collagen I and collagen III normalised to the housekeeping gene RPL4 were displayed as the mean of 2^−∆Ct^ ± standard deviation.

### 2.5. Masson–Goldner Trichrome Staining

Connective tissue in myocardial samples was stained by Masson–Goldner trichrome staining. All staining solutions were purchased from Morphisto GmbH (Offenbach am Main, Germany). Histological sections of 3 µm thickness were deparaffinized in xylole and hydrated in 96%, 80%, 70% and 60% ethanol and distilled water. Sections were stained with Weigert’s iron hematoxylin for 15 min followed by washing step with distilled water and blueing in non-distilled water for 8 min. Cytoplasma was stained with Goldner I for 4 min followed by incubation in the staining solutions Goldner II for 30 min and Goldner III for 6 min. Then, sections were rinsed in acetic acid for 2 min and non-distilled water for 1 min followed by dehydration in 96% ethanol, isopropanol and xylole. Stained sections were mounted with Entellan^®^ (Merck KGaA, Darmstadt, Germany). Microscopic evaluation was performed with the Axioplan 2 microscope and the Axio Vision Release 4.8.23 Sp3 software (both Carl Zeiss AG, Oberkochen, Germany). The percentage of the green-stained area (=connective tissue) to the total area of each section was quantified by Photoshop CS2 (Adobe Inc., San Jose, CA, USA).

### 2.6. Immunohistochemistry of Collagen I and Collagen III

Collagen I and collagen III protein expression were quantified by immunohistochemistry in myocardial sections as described previously [5]. Deparaffinized and rehydrated sections were cooked in 0.01 M sodium citrate (pH 6.0) for 30 min. Following cooling, endogenous peroxidases were blocked with 60% methanol/40% tris-buffered saline (TBS)/0.1% H_2_O_2_ for 50 min. Sections were rinsed 3 times in TBS and blocked with 2% bovine serum albumin (BSA)/TBS for 60 min. Primary antibodies for collagen I (Abcam, Cambridge, UK) and collagen III (Cell signalling Technology^®^, Boston, MA, USA) were added to the sections and incubated overnight at 4 °C. Primary antibodies were rinsed 3 times with TBS before secondary antibody incubation with anti-rabbit biotin (Sigma Aldrich, St. Louis, MO, USA) was started for 60 min. Following secondary antibody incubation, the sections were rinsed 3 times in TBS, incubated with streptavidin–horseradish peroxidase (Thermo Fisher Scientific) for 60 min, rinsed 3 times in TBS, incubated in AEC staining solution (Sigma Aldrich) for 10 min, rinsed in TBS and counterstained with hemalum. Stained sections were mounted in glycerol gelatine. Microscopic evaluation was performed with the Axioplan 2 microscope and the Axio Vision Release 4.8.23 Sp3 software (both Carl Zeiss AG, Oberkochen, Germany). The percentage of the green-stained area (=connective tissue) to the total area of each section was quantified by Photoshop CS2 (Adobe Inc., San Jose, CA, USA). Microscopic evaluation was performed as described for Masson–Goldner trichrome staining. The percentage of AEC-positive stained area (=collagen-positive) to the total area of each section was quantified by Photoshop CS2 (Adobe Inc., San Jose, CA, USA).

### 2.7. Statistics

Data were collected using Microsoft Excel 2016 (Microsoft Corporation, Redmond, WA, USA). Statistical analyses were performed using SPSS version 28 (IBM Corp., Armonk, NY, USA). Continuous variables are presented as mean ± standard error of the mean, categorical variables are represented as the number (percent). Group comparisons of metric data were performed with t tests. The Levene test was used to analyse homoscedasticity. Group comparisons of ordinal data were performed using the χ^2^ test. In case of expected frequencies < 5 and degree of freedom > 1, the Fishers’s exact test was applied. In case of expected frequencies < 5 and degree of freedom = 1, the Yates correction was performed. *p* values ≤ 0.05 were acknowledged as significant.

## 3. Results

### 3.1. Demographic and Clinical Characteristics

Patients of the recovery group recovered within 17 months [95% CI: 4.8–28.8] and were weaned from LVAD. Patients were comparable regarding the left ventricular ejection fraction (*p* = 0.32), NT-proBNP serum levels (*p* = 0.19) and comorbidities such as arterial hypertension (*p* = 0.38), coronary heart disease (*p* = 0.36), type 2 diabetes (*p* = 0.22) and kidney function (estimated glomerular filtration rate *p* = 0.11; creatinine levels *p* = 0.32) prior to LVAD implantation (Table 1).

### 3.2. mRNA Expression of Collagen Type I and III

Myocardial tissue was analysed for collagen I and collagen III expression. mRNA expression analysis could not detect a difference in collagen I (non-recovery group: 0.14 [95% CI: 0.05–0.24], recovery group: 0.72 [95% CI: −0.55–1.99], *p* = 0.16) and collagen III expression (non-recovery group: 0.42 [95% CI: 0.29–0.54], recovery group: 1.07 [95% CI: −0.21–2.34], *p* = 0.12) between the non-recovery and the recovery group.

### 3.3. Histological and Immunohistochemical Evaluation

**Figure 1:**
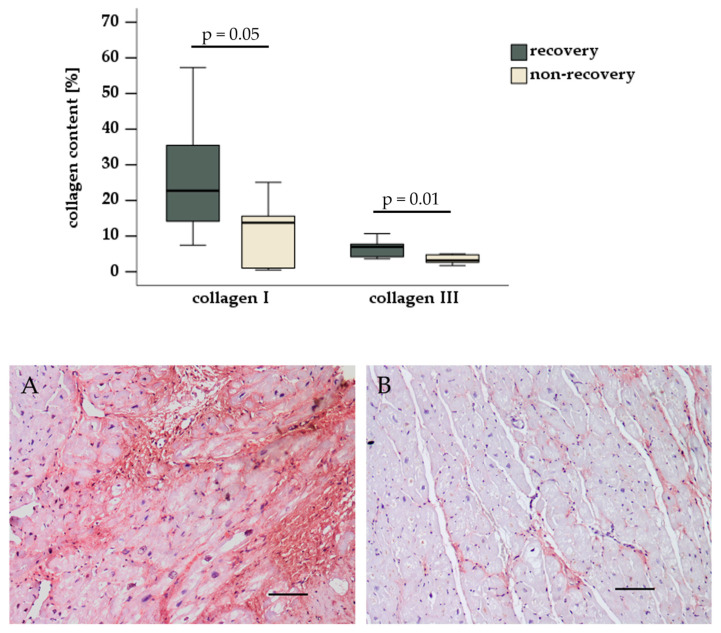
Collagen I and III content in the pre-implant left ventricle. The collagen I (**A**) and collagen III (**B**) content was quantified following immunohistochemical staining of myocardial tissue. Scale bar = 100 µm.

The histological evaluation revealed comparable values for the percentage of total connective tissue stained by Masson–Goldner trichrome staining (non-recovery: 46.3% [95% CI: 15.9–76.7], recovery: 43.4% [95% CI: 13.7–73.2], *p* = 0.43). Immunohistochemical staining revealed that the percentages of collagen I (non-recovery: 11.6% [95% CI: 1.7–21.5], recovery: 29.6% [95% CI: 8.0–45.3], *p* = 0.05) and of collagen III (non-recovery: 3.4% [95% CI: 2.1–4.7], recovery: 6.7% [95% CI: 4.0–9.4], *p* = 0.01) were reduced in patients who do not recover compared to patients who recover under LVAD support Figure 1.

## 4. Discussion

Our data indicate that histological evaluation for collagen expression prior to LVAD implantation may be a useful tool for detecting differences in the collagen I and collagen III content of left ventricle tissue. This evaluation could potentially assist in predicting weaning success and, consequently, the treatment goal of LVAD support in patients with heart failure.

The quantification of collagens in the myocardium of LVAD patients started since LVAD of the first generation had been implanted [6,7,8]. The majority of studies have documented alterations in collagen content or fibrosis both prior to and following LVAD implantation. However, investigations in successfully weaned patients are rare. Collagens are a subset of ECM proteins that form a structural framework referred to as a “fibre scaffold”. This framework is vital for preserving the geometry and facilitating the transmission of force within the myocardial cells that are embedded within it. It is reasonable to hypothesise that the recovery of the myocardium with LVAD support may be hindered, or even rendered impossible, in cases of the advanced destruction or remodelling of the ECM scaffold. Therefore, it is imperative that there is sufficient expression of collagen I to preserve cardiac geometry, structure and physiological stiffness, as well as of collagen III to form a thin network that increases tissue elasticity [9]. These are requirements for myocardial recovery. The present hypothesis is supported by the observation that LVAD support has been demonstrated to result in increased collagen expression, which follows a biphasic pattern with differential collagen expression profiles [10,11].

Heart failure is associated with an imbalanced matrix collagen content, and LVAD support has been shown to increase or decrease collagen subtypes [12]. Myocardial stiffness and fibrosis are the result of an imbalance and excessive accumulation of ECM proteins [13,14]. Conversely, overactive matrix-degrading processes promote diastolic remodelling and counteract myocardial recovery. It is evident that a balanced ECM is of paramount importance in providing the structural support and ventricular geometry necessary for myocardial recovery. It is the contention of the present study that successful myocardial recovery is contingent upon adequate ventricular geometry prior to LVAD implantation. Consequently, the estimation of proteins that ensure ventricular geometry and structural support in the myocardium could be an important element in evaluating the probability of weaning success and defining the treatment goal of LVAD support in heart failure patients.

The study is limited by the low number of patients. Myocardial recovery in end-stage heart failure patients with LVAD support is not a common event. Thus, sampling of myocardial tissue from recovery patients is time-consuming and extends over a longer period. Genetic testing for DCM has not been performed. Consequently, the process of sampling myocardial tissue from recovery patients is both time-consuming and protracted. Genetic testing for DCM has not been performed. Therefore, the existence of genetic mutations in this small study cohort that could influence collagen I and III content remains unclear. Tissue sampling was performed in the coring region of the apex of the left ventricle. It is important to note that sampling of a single region in the left ventricle may not be representative of the entire structure, raising concerns about the validity of the results obtained from such an approach.

The quantification of overall ECM or connective tissue using Masson–Goldner trichrome staining did not differ between recovery and non-recovery LVAD patients. Thus, we recommend the quantification of collagen I and collagen III expression of the left ventricular core within the scope of LVAD implantation by immunohistology to provide further information on the LVAD weaning probability.

## Figures and Tables

**Table 1:** Demographic and clinical characteristics of recovery and non-recovery patients.

	Recovery Group *n* = 7	Non-Recovery Group *n* = 7	*p* Value
**Male gender**	2 (29%)	2 (29%)	1
**Age at LVAD implantation [yrs]**	57.0 ± 9.0	61.7 ± 6.4	0.14
**BMI [kg/m^2^]**	31.2 ± 7.5	27.1 ± 6.5	0.15
**LVEF [%]**	18.4 ± 7.3	16.4 ± 7.9	0.32
**NT-proBNP [ng/L]**	4619 ± 4120	7102 ± 3319	0.19
**NYHA classification** Class III Class IV	5 (71%) 2 (29%)	2 (29%) 5 (71%)	0.29
**Nicotine abuse** Smoker Non-smoker Ex-smoker	0 (0%) 5 (71%) 2 (29%)	0 (0%) 5 (71%) 2 (29%)	1
**Arterial hypertension**	4 (57%)	5 (71%)	1
**Coronary heart disease**	2 (29%)	0 (0%)	0.45
**Type 2 diabetes**	3 (43%)	2 (29%)	1
**Renal function** eGFR [mL/min/1.73 m^2^] Creatinine [µmol/L]	57.4 ± 26.0 131 ± 90	41.1 ± 19.8 151 ± 64	0.11 0.32

Footnote Table 1: BMI: body mass index; LVEF: left ventricular ejection fraction; eGFR, estimated glomerular filtration rate; NT-proBNP, N-terminal pro b-type natriuretic peptide; NYHA: New York Heart Association.

## Data Availability

The data presented in this study are available on request from the corresponding authors due to ethical permission.
